# Sacituzumab govitecan combined with tislelizumab as second-line therapy for recurrent or metastatic cervical cancer: a case report and literature review

**DOI:** 10.1186/s12905-026-04481-4

**Published:** 2026-04-22

**Authors:** Hui Li, Miaofang Wu, Yichun Xing, Jing Li, Zhongqiu Lin, Dongyan Wang

**Affiliations:** 1 Department of Gynecologic Oncology, Sun yat-sen Memorial Hospital, Sun yat-sen University, Guangzhou, Guangdong China; 2 Guangdong Provincial Clinical Research Center for Obstetrical and Gynecological Diseases, Guangzhou, Guangdong China

**Keywords:** Sacituzumab govitecan, Tislelizumab, Recurrent or metastatic cervical cancer, Second-line therapy, Case report

## Abstract

**Background:**

The second-line treatment for recurrent or metastatic cervical cancer remains uncertain and controversial. Sacituzumab govitecan, the first-in-class antibody-drug conjugate (ADC) targeting trophoblast cell surface antigen 2 (TROP2), has demonstrated promising efficacy and safety in several clinical trials for cervical cancer. However, real-world clinical data on sacituzumab govitecan combined with immunotherapy remains limited.

**Case presentation:**

A 73-year-old woman diagnosed with stage IIIC1r (FIGO 2018) cervical squamous cell carcinoma underwent concurrent chemoradiotherapy as the primary treatment. The disease recurred 20 months later; thus 3 cycles of chemotherapy combined with bevacizumab and additional 5 cycles of bevacizumab monotherapy were recommended. Although the patient achieved complete response in both the primary treatment and the first recurrence treatment, the second recurrence was detected 15 months later. The patient underwent 6 cycles of sacituzumab govitecan combined with tislelizumab and additional 6 cycles of tislelizumab monotherapy. Partial response was achieved with an 88.1% reduction in target lesion size, along with a PFS of 9 months and an ongoing OS of 13 months.

**Conclusions:**

Sacituzumab govitecan combined with tislelizumab demonstrated potential treatment response in this case. However, further prospective clinical trials are warranted to validate its efficacy and safety in the difficult second-line setting of recurrent or metastatic cervical cancer.

**Supplementary Information:**

The online version contains supplementary material available at 10.1186/s12905-026-04481-4.

## Background

Cervical cancer is the fourth most common malignancy in females worldwide, with approximately 85% of cases occurring in low- and middle-income countries [1, 2]. In 2022, China reported 150,000 new cases and 56,000 deaths attributable to cervical cancer [3]. Currently, the combination of pembrolizumab and chemotherapy, with or without bevacizumab, is the preferred first-line regimen for programmed death-ligand 1 (PD-L1)-positive recurrent or metastatic cervical cancer (R/M CC) [4]. However, a significant proportion of patients eventually experience disease progression and require subsequent treatment. Yet, the second-line treatment with single-agent chemotherapy remains unsatisfactory, exhibiting limited efficacy with an objective response rate (ORR) of approximately 5% and a median PFS of only 3 months [5–7]. While National Comprehensive Cancer Network (NCCN) guidelines recommend pembrolizumab as the preferred second-line option for R/M CC patients with TMB-H/PD-L1 positive/MSI-H/dMMR tumors, the clinical gains remain modest [4]. Although PD-1 inhibitors have improved the ORR compared to single-agent chemotherapy (16.4% vs. 6.3%) in the second-line treatment of R/M CC, the median OS benefit of 4.6 months highlights the urgent need for novel therapeutic approaches [7] .

In recent years, antibody-drug conjugate (ADC) has demonstrated promising efficacy and safety across multiple solid tumors, including cervical cancer. And NCCN guidelines have also recommended tisotumab vedotin (TV) as the preferred second-line option for R/M CC patients, and trastuzumab deruxtecan (T-DXd) is recommended for human epidermal growth factor receptor 2 (HER2)-overexpressing (immunohistochemistry [IHC] 3 + or 2+) cervical cancer [4]. The ADC landscape continues to evolve with ongoing clinical trials investigating novel molecular targets, such as Trophoblast cell surface antigen 2 (TROP2) [8]. TROP2 is overexpressed in multiple malignancies and promotes tumorigenesis, invasion and metastatic dissemination. Previous study has found that TROP2 was positive in 88.7% cervical cancer patients, and TROP2 expression was significantly associated with decreased PFS and OS [9]. Sacituzumab govitecan (SG) is a first-in-class TROP2-targeting monoclonal antibody conjugated to SN-38, which is an active metabolite of the topoisomerase I inhibitor irinotecan. The phase I/II IMMU-132-01 basket trial demonstrated manageable safety profile and promising efficacy on several epithelial cancers [10]. Moreover, the phase III ASCENT trial enrolling metastatic triple-negative breast cancer has also showed that SG significantly improved patients’ survival and nearly doubled the median OS compared to chemotherapy (12.1 vs. 6.7 months) [11]. The U.S. Food and Drug Administration (FDA) has approved SG for metastatic triple-negative breast cancer, hormone receptor-positive/HER2-negative breast cancer, and metastatic urothelial carcinoma [12]. Preclinical studies have demonstrated the potent antitumor activity of SG against TROP2-positive cervical cancer cell lines [13]. The EVER-132-003 phase II basket trial’s interim analysis reported a remarkable 50% ORR among 18 R/M CC patients [14]. Despite these encouraging results, real-world clinical data on SG in R/M CC remain sparse.

On the other hand, a real-world study has demonstrated that the combination of ADC and immune checkpoint inhibitor (ICI) has improved ORR and survival over ADC monotherapy in metastatic gastric cancer [15]. And theoretically, the combination therapy might produce synergistic effect to overcome resistance to either ADC or ICI monotherapy [16]. Herein, we present a case of R/M CC achieving partial response with a combination of SG and tislelizumab as the second-line treatment. In addition, we provide a narrative review of the literature regarding the potential synergistic mechanism of the combination therapy of TROP2 ADC and ICI.

## Case presentation

**Fig. 1:**
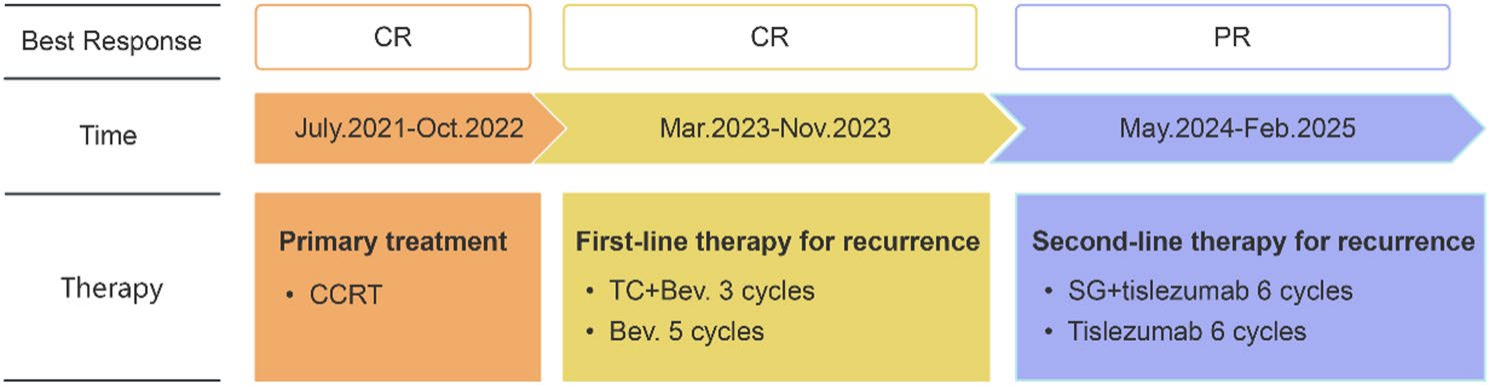
Timeline and the best response according to Response Evaluation Criteria in Solid Tumors version 1.1 of the patient’s treatments. CR: complete response; PR: partial response; CCRT: concurrent chemoradiotherapy; T: paclitaxel; C: carboplatin; Bev: bevacizumab; SG: sacituzumab govitecan

In July 2021, a 73-year-old woman complained of abnormal vaginal bleeding, and was subsequently diagnosed with stage IIIC1r (FIGO 2018) squamous cell carcinoma of the cervix. She underwent definitive concurrent chemoradiotherapy at a local hospital, and achieved complete response (CR, according to Response Evaluation Criteria in Solid Tumors version 1.1, RECIST version 1.1). After the completion of primary therapy, she was followed up by routine surveillance (Fig. 1). In March 2023, pulmonary and mediastinal lymph node metastases were detected by CT scan, and serum squamous cell carcinoma antigen (SCCA) level was elevated, indicating disease recurrence. The patient was subsequently initiated on a triplet regimen consisting of three cycles of paclitaxel, carboplatin, and bevacizumab, followed by five cycles of bevacizumab maintenance therapy. A second CR was achieved; however, the patient elected to discontinue further treatment at that time.

**Fig. 2:**
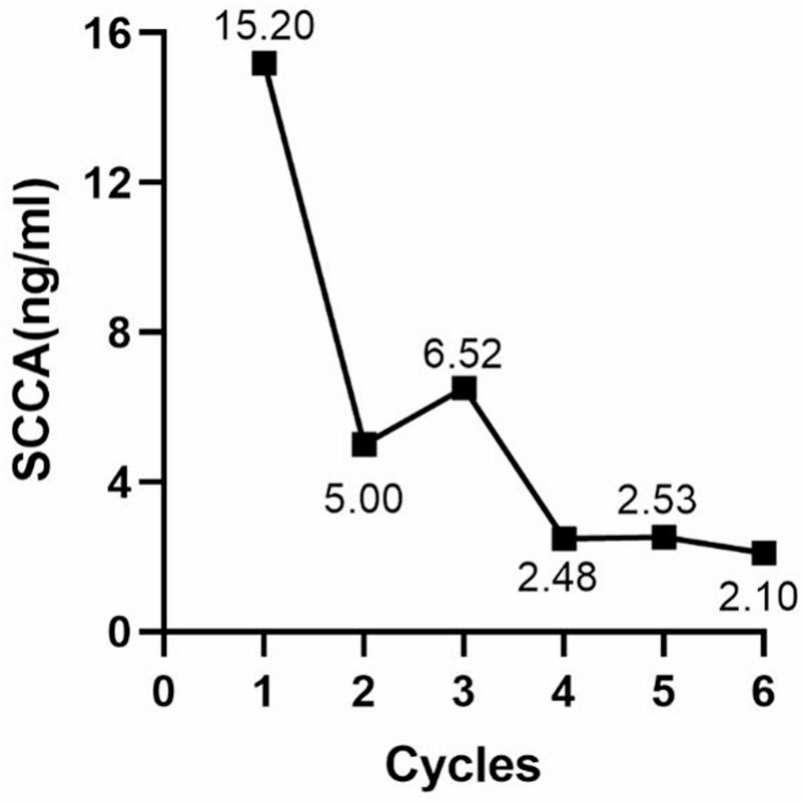
Changes in serum squamous cell carcinoma antigen (SCCA) levels during the second-line therapy of the R/M CC case

**Fig. 3:**
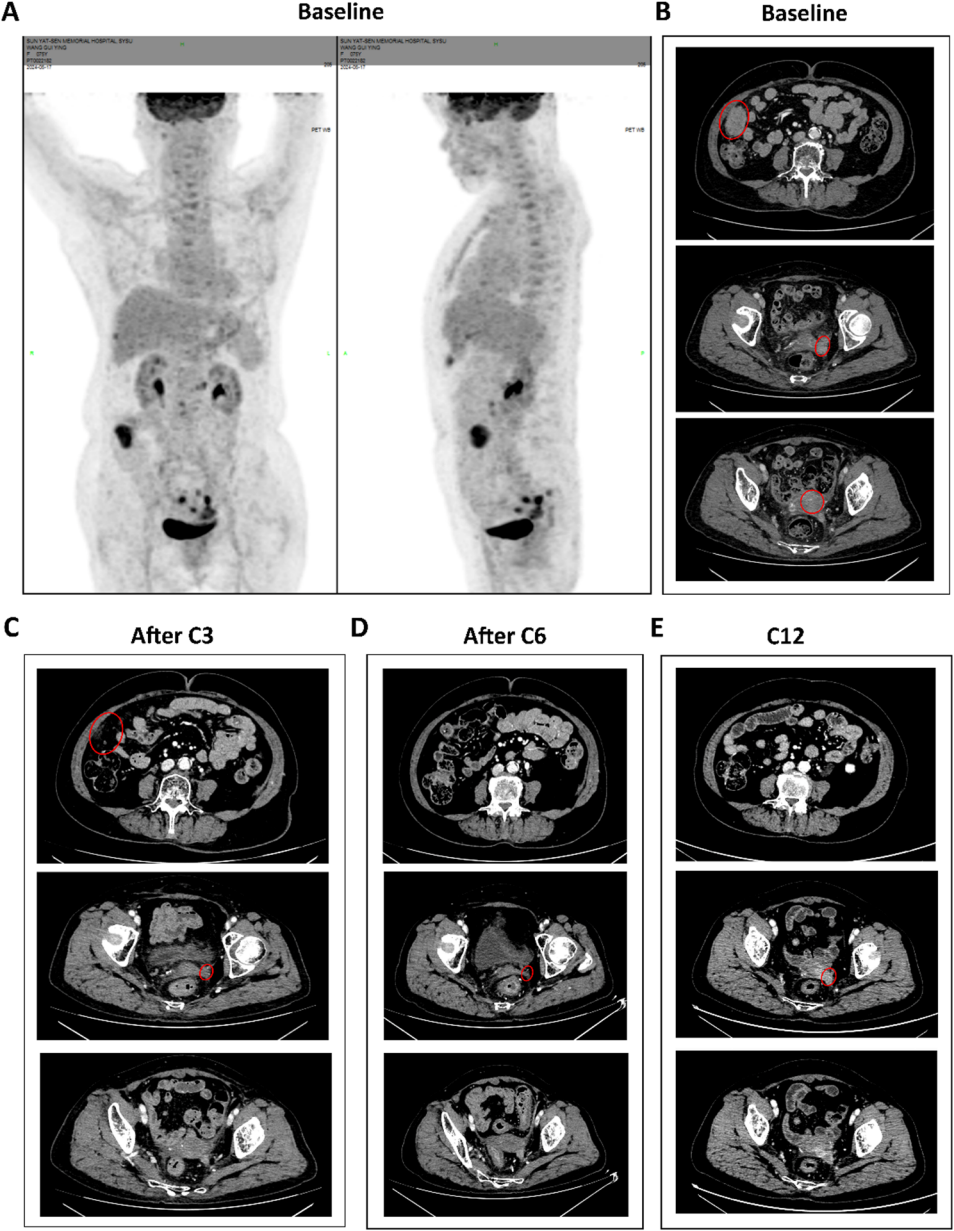
Imaging evaluation during the second-line treatment of the case according to Response Evaluation Criteria in Solid Tumors version 1.1. **A** PET-CT demonstrated multiple metabolically active lesions involving the whole body. **B**-**E** CT scans of target lesions from top to bottom: right abdomen, pelvis and cervix. **B** Baseline assessment. **C** Efficacy assessment after C3 (cycle 3) demonstrated a partial response. **D** Efficacy assessment after C6 (cycle 6) demonstrated a partial response. **E** Efficacy assessment on C12 (cycle 12) demonstrated disease progression with enlargement of the pelvic lesion

**Fig. 4:**
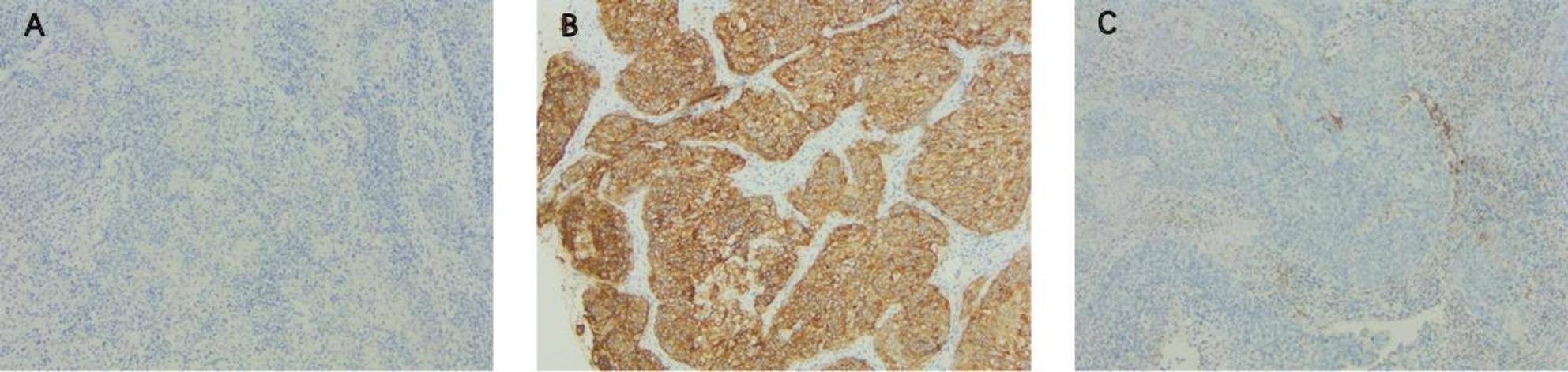
Immunohistochemistry of HER2 (human epidermal growth factor receptor 2), TROP2 (trophoblast cell surface antigen 2) and PD-L1 (programmed cell death-ligand 1) CPS (Combined Positive Score). **A** HER2 (0). **B** TROP2 (90%, 3+). **C** CPS = 3 (PD-L1 [22C3], PD-L1 [Neg] [-])

In May 2024, the patient came to our hospital with elevated SCCA levels (15.2 ng/ml) (Fig. 2). A subsequent PET/CT scan revealed a multi-focal second recurrence involving the cervical remnant, peritoneal carcinomatosis (including right abdominal and pelvic lesions), and para-aortoiliac lymphadenopathy (Fig. 3A). Histopathological re-evaluation of archival formalin-fixed, paraffin-embedded tumor tissue from the initial biopsy confirmed moderately to poorly differentiated squamous cell carcinoma. Immunohistochemical profiling revealed TROP2 overexpression (90% membranous staining, 3 + intensity), a PD-L1 Combined Positive Score (CPS) of 3, and HER2 negative (HER2 0) (Fig. 4).

Following a multidisciplinary tumor board review, combination therapy of SG (10 mg/kg on days 1 and 8) and tislelizumab (200 mg on day 1) every 3 weeks was recommended based on TROP2 overexpression and PD-L1 positivity. We thoroughly discussed the potential benefits and risks of this off-label combination therapy with the patient, and written informed consent was acquired. During the treatment period, tumor response was assessed by CT scans every 3 to 6 months, and evaluated according to RECIST version 1.1 as routinely performed in the standard of care of oncology clinic. After 3 cycles, treatment assessment demonstrated a partial response (PR), with target lesions reduced by 70.3% and SCCA decreased to 2.48 ng/ml. The best overall response was achieved after 6 cycles, with an 88.1% reduction in target lesion size and SCCA decreased to 2.10 ng/ml (Figs. 2 and 3B-D; Table 1).

**Table 1:** Efficacy assessment according to Response Evaluation Criteria in Solid Tumors version 1.1 during second-line treatment of the case

Cycle	Target lesions (mm)						Non-target lesions	
	Right abdomen	Pelvic	Cervix	Sum	Change Ratio	Efficacy	Para-aortoiliac LNs	Efficacy
Baseline	44	36	38	118	-	-	Exist	-
After C3	21	14	0	35	-70.3%	PR	Exist	Non-CR/Non-PD
After C6	0	14	0	14	-88.1%	PR	Exist	Non-CR/Non-PD
After C12	0	38	0	38	171.4%	PD	Exist	Non-CR/Non-PD

*Cycle* Chemotherapy cycle, *C3* Cycle 3, *C6* cycle 6, *C12* Cycle12, *LNs* Lymph nodes, *PR* Partial response, *CR* Complete response, *PD* Progressive disease

Following the first cycle of combination therapy, the patient suffered afebrile Grade 4 neutropenia (CTCAE v5.0) on day 12. Laboratory results indicated a white blood cell count of 0.77 × 10⁹/L and neutrophil count of 0.14 × 10⁹/L. The patient was hospitalized and treated with daily subcutaneous short-acting granulocyte colony-stimulating factor (G-CSF; 200 µg) for six days until leukocyte and neutrophil counts returned to normal level. Antibiotic therapy was withheld as the patient remained afebrile. The second treatment cycle was delayed by one day. We believed neutropenia can be prevented by prophylactic G-CSF support strategy in further combination therapy. After thorough discussion with the patient and her families, we attained their consent and chose to continue the treatment without dose reduction. Subsequent treatment cycles adopted a prophylactic G-CSF support strategy, which consisted of daily short-acting G-CSF injections for 3 consecutive days starting on day 4 after each D1 administration of SG + tislelizumab, followed by a single dose of long-acting G-CSF 24 h after the completion of SG on day 8. No further grade 3 or higher neutropenia occurred, and the patient completed the following treatment cycles on time. During the treatment course, the patient also developed Grade 1 hypothyroidism, which was successfully managed with levothyroxine. After cycle 6, we recommended continuing the treatment with a 25% dose reduction in SG given the drug’s perceived efficacy and toxicity. However, after careful consideration, the patient declined the dose modification and opted for permanent discontinuation of SG mainly due to the unbearable financial burden besides cumulative hematologic toxicity.

Six more cycles of tislelizumab monotherapy were continued until disease progression with an SCCA rebound (9.47 ng/ml) and radiographic enlargement of the pelvic target lesion (Fig. 3E; Table 1). We recommended brachytherapy with continued tislelizumab, but the patient declined further intravenous medication and was subsequently referred to the radiation oncology department for brachytherapy. Overall, the patient achieved a PFS of 9 months and an ongoing OS of 13 months at the last follow-up on July 7, 2025.

## Discussion and Conclusions

In this article, we present a case of R/M CC treated by the combination of SG and tislelizumab as the second-line therapy. We specifically recommended the combination treatment due to the positivity of TROP2 expression and PD-L1, as well as the preserved performance status of the patient. The patient achieved PR after the second-line therapy with a PFS of 9 months and an ongoing OS of 13 months at the last follow-up. Currently, effective and safe second-line therapy for R/M CC patients has not been established. The recommended second-line treatment of pembrolizumab or tisotumab vedotin only slightly improved the median PFS compared to single-agent chemotherapy (4 months vs. 3 months), highlighting the urgent need for novel therapeutic approaches [4, 6, 17]. This case report provides a precedent of the combination of SG and tislelizumab as the second-line therapy for R/M CC, which also leads to the synergistic hypothesis between antibody-drug conjugate (ADC) and immune checkpoint inhibitor (ICI).

SG targets the TROP2 expressing tumor cells with sacituzumab, and release its topoisomerase I inhibitor payload govitecan through the hydrolysis of the ADC linker. Furthermore, free govitecan is membrane-permeable and capable of eliciting anti-tumor effect in adjacent tumor cells regardless of their TROP2 expression status (bystander effect) [18, 19]. Ongoing clinical trials have been investigating the efficacy of TROP2 ADCs, including SG, in R/M CC [20–22]. In the interim analysis of the basket trial EVER-132-003, the ORR of SG monotherapy for R/M CC was 50%, the median PFS was 8.1 months, the 6 months PFS rate was 58% [14]. This result indicated that SG may have better efficacy than traditional chemotherapy in the treatment of R/M CC. Preclinical evidence suggests a positive correlation between TROP2 expression intensity and the antitumor activity of SG [23]. In this case, immunohistochemical profiling revealed exceptionally high TROP2 expression (90% membranous staining, 3 + intensity), which provided a strong molecular rationale for the application of SG.

On the other hand, ICI has demonstrated modest but clinically meaningful efficacy in the second-line treatment of R/M CC. The phase III EMPOWER-Cervical 1 trial has established the superiority of cemiplimab (a PD-1 antibody) over investigator’s choice chemotherapy, reporting a significantly higher ORR (16.4% vs. 6.3%) and a median overall survival (OS) improvement of 4.6 months [7]. These findings are complemented by the Phase II KEYNOTE-158 trial, where pembrolizumab monotherapy achieved an ORR of 12.2% in a heavily pre-treated cohort [24]. Similarly, in the phase I/II CheckMate 358 trial, nivolumab monotherapy achieved an ORR of 26.3% among 19 R/M CC patients with a median OS of 21.9 months [25]. Tislelizumab is a programmed cell death protein 1 (PD-1) inhibitor, which has high affinity for PD-1 with a 100-fold slower off-rate than pembrolizumab. For R/M CC, tislelizumab monotherapy demonstrated similar ORR and median PFS compared to pembrolizumab monotherapy [26]. In addition, tislelizumab is more affordable and cost-effective compared to pembrolizumab for Chinese patients due to its coverage by national medical insurance.

Theoretically, the combination of SG and tislelizumab leverages a multifaceted synergy that addresses several levels of immune evasion [16]. Initially, the IgG1 backbone of the ADC may stimulate the innate immune response via antibody-dependent cellular cytotoxicity, recruiting natural killer cells and macrophages to the tumor microenvironment (TME). In addition, the cytotoxic payload, SN-38, induces Immunogenic Cell Death. This process triggers the release of tumor-associated antigens and damage-associated molecular patterns, which are essential for dendritic cell maturation and the subsequent “priming” of the adaptive immune response. Moreover, the inflammation induced by the ADC also leads to PD-L1 upregulation, which is the primary target of the ICI. By converting the “cold” immune-excluded tumors into “hot” immune-infiltrated tumors, the ADC sensitizes the TME to the reinvigoration of tumor-reactive cytotoxic T lymphocytes by the ICI, and unlocks the immune response against tumor cells. Therefore, ADCs combined with immunotherapy may be able to overcome resistance to either single agent therapy, providing a robust treatment option even in heavily pre-treated R/M CC.

A narrative literature review on the combination therapy of TROP2 ADC and ICI for various solid tumors was performed by a query search of PubMed database (supplementary material), and a manual search of the ASCO and the ESMO conference abstracts of the recent 5 years. Studies utilizing the combination therapy of TROP2 ADC and ICI to treat cancer patients were included, and review articles were excluded. In total, 4 phase II clinical trials were identified (Table 2 ) [20, 27–29]. Notably, a phase II trial enrolling 38 R/M CC patients achieved a remarkable ORR of 57.9% with sacituzumab tirumotecan (TROP2 ADC) and pembrolizumab in the second-line setting. Although the median PFS was not reached with a median follow-up time of 6.2 months, this trial along with our case have suggested that the combination therapy of TROP2 ADC and PD-1 antibody was a potential option for the second-line treatment of R/M CC. However, future Phase III randomized controlled trials are warranted to validate these findings and to establish the combination therapy as a new standard of care for this difficult situation.

The identification of reliable predictive biomarkers remains a critical challenge in personalizing ADC-ICI combination therapy. Evidence from two trials on metastatic lung cancer have both demonstrated that the efficacy of the combination therapy was seemingly correlated with PD-L1 level. Specifically, patients with higher PD-L1 TPS level had better ORR and longer PFS when treated by the combination therapy of TROP2 ADC and ICI (Table 2). Our case was also PD-L1 positive (CPS = 3) and achieved partial response with the combination therapy, suggesting that PD-L1 level might also be a potential predictive marker for the combination therapy of TROP2 ADC and ICI in R/M CC. However, it should be noted that PD-L1 TPS level from lung cancer trials cannot be converted into the CPS level in cervical cancer patients. Consequently, prospective clinical trials specifically designed for the R/M CC population are required to validate the predictive value of the PD-L1 CPS level. Furthermore, the role of TROP2 expression as a predictive biomarker for ADC-ICI combinations remains paradoxical. While TROP2 overexpression was a defining feature of our patient (IHC 3+, 90%), data from the EVOKE-02 trial did not demonstrate a clear correlation between TROP2 expression levels and clinical efficacy [28]. Similarly, the predictive utility of other markers, such as tumor mutational burden (TMB), remains under-investigated in this context, highlighting the need for further translational research.

**Table 2:** Clinical trials of TROP2 ADC combined with ICI in various solid tumors

	Wang et al [20]		Grivas et al [27]	Reck et al [28]			Fang et al [29]		
Cancer	Recurrent/metastatic cervical cancer		Metastatic urothelial cancer	Metastatic non-small cell lung cancer			Metastatic non-small cell lung cancer		
Treatment line	Second-line		Second-line	First-line			First-line		
ADC	Sacituzumab tirumotecan		Sacituzumab govitecan	Sacituzumab govitecan			Sacituzumab tirumotecan		
ICI	Pembrolizumab		Pembrolizumab	Pembrolizumab			Tagitanlimab		
Patients number	38		41	92			81		
				PD-L1 TPS < 50%	PD-L1 TPS ≥ 50%		PD-L1 TPS < 1%	PD-L1 TPS ≥ 1%	PD-L1 TPS ≥ 50%
ORR	0.579		0.41	0.29	0.67		0.471	0.681	0.778
Median PFS	NA		5.3	7	13.1		12.4	17.8	17.8
Median OS	NA		12.7	NA			NA		
Most common Grade ≥ 3 AE (occurrence rate)	Neutropenia (23%)	Neutropenia (37%)		Neutropenia (17%)		Neutropenia (45%)			

*ADC* Antibody-drug conjugate, *ICI* Immune checkpoint inhibitor, *PD-L1* Programmed cell death-ligand 1, *TPS* Tumor proportion score, *ORR* Objective response rate, *PFS* Progression-free survival, *OS* Overall survival, *AE* Adverse events

The safety profiles reported across the identified clinical trials were consistent and manageable, with no novel treatment-related adverse events compared to historical ICI and chemotherapy trials. The most common grade ≥ 3 treatment-related adverse event was neutropenia across all 4 trials. Considering the approximate 50% occurrence rate of neutropenia in SG monotherapy trials [11, 14], the combination therapy seemingly did not increase the incidence of neutropenia. However, prospective trials with larger cohorts remain necessary to validate the safety profile of the combination therapy. In our case, the patient suffered grade 4 neutropenia and grade 1 hypothyroidism, which were managed by granulocyte colony-stimulating factor and levothyroxine respectively. Considering the prevalence and severity of neutropenia in the combination therapy, prophylactic G-CSF support strategy should be considered, especially for elderly or potentially frail patients.

Other ADCs targeting different antigens combined with ICI have also demonstrated promising efficacy in various solid tumors. A multicenter real-world study has shown that RC48 (HER2 ADC) combined with ICIs for locally advanced bladder cancer achieved an unprecedented 100% ORR and significantly prolonged radiographic PFS (rPFS) [30]. The AK001 study reported that RC48 combined with cadonilimab (PD-1/CTLA-4 bispecific antibody) as second-line therapy for R/M CC achieved an ORR of 50% [31]. The EV-103 phase Ib/II study has also demonstrated that enfortumab vedotin (nectin-4 ADC, EV) combined with pembrolizumab achieved higher ORR than EV monotherapy with a similar safety profile [32]. We believe ADC with different antigen targets might also exert a synergistic anti-tumor activity with ICI, and numerous combinations are promising [16]. The various combination strategies of ADC and ICI might provide reasonable options for the difficult second-line setting of R/M CC. However, further investigation is warranted to validate the efficacy and safety of these combinations, and effective prediction biomarkers are urgently needed in the era of personalized medicine.

Affordability is an important aspect of treatment strategy in clinical practice. Although tislelizumab was covered by national medical insurance, SG was not. The decision to discontinue the combination therapy and switch to tislelizumab monotherapy in this case was believed to be made mainly due to the financial toxicity of SG, considering the successful management of the neutropenia. The patient had spent approximately USD 30,000 on SG alone; it had become a tremendous financial burden for the patient and her family. Thus, cutting the price of SG or coverage by the insurance should be considered to reduce the heavy financial burden of cancer patients and improve treatment adherence.

Several limitations on this case report should be mentioned. First, the exceptional response observed in this patient may be subject to selection bias. We specifically recommended this treatment due to the positivity of TROP2 expression and PD-L1, as well as the preserved performance status of the patient. Therefore, this combination therapy might not be generalizable for all R/M CC patients in the second-line setting. Second, the patient received 6 more cycles of ICI monotherapy after 6 cycles of combination therapy before disease progression due to toxicity and financial burden. This complicated the attribution of PFS and OS benefits, and further prospective clinical trials are needed to compare the combination therapy with the ICI monotherapy in the second-line setting of R/M CC patients. Finally, the inherent limitation of this single-patient case report without comparator and with limited follow-up duration should be noted. While our findings provide a potential clinical signal, they should be interpreted as hypothesis-generating rather than definitive. Large-scale, prospective randomized controlled trials are required to compare the efficacy and safety of the combination therapy against established monotherapies in the second-line setting for R/M CC.

## Conclusion

In summary, SG combined with tislelizumab demonstrated potential treatment response and manageable safety profile in this case. The hypothetic synergy between TROP2 ADC and ICI is promising but require further validation. Further prospective clinical trials incorporating predictive biomarkers analysis are warranted to evaluate its efficacy and safety in the difficult second-line setting for R/M CC patients.

## Supplementary Information

Supplementary Material 1.

Supplementary Material 2.

## Data Availability

No datasets were generated or analysed during the current study.

## Abbreviations

ADC: Antibody-drug Conjugate
AE: Adverse Event
ASCO: American Society of Clinical Oncology
CPS: Combined Positive Score
CR: Complete Response
ESMO: European Society For Medical Oncology
FDA: Food and Drug Administration
HER2: Human Epidermal Growth Factor Receptor 2
ICI: Immune Checkpoint Inhibitor
IHC: Immunohistochemistry
MHC-I: Major Histocompatibility Complex Class I
NCCN: National Comprehensive Cancer Network
ORR: Objective Response Rate
OS: Overall Survival
PD: Progressive Disease
PD-L1: Programmed Death-Ligand 1
PFS: Progression-free Survival
PR: Partial Response
R/M CC: Recurrent or Metastatic Cervical Cancer
RECIST version 1.1: Response Evaluation Criteria in Solid Tumors version 1.1
SCCA: Serum Squamous Cell Carcinoma Antigen
SG: Sacituzumab Govitecan
T-DXd: Trastuzumab Deruxtecan
TPS: Tumor Proportion Score
TROP2: Trophoblast Cell Surface Antigen 2
TV: Tisotumab Vedotin

## References

[1] Bray F, Laversanne M, Sung H, et al. Global cancer statistics 2022: GLOBOCAN estimates of incidence and mortality worldwide for 36 cancers in 185 countries. CA Cancer J Clin. 2024;74:229–63. 10.3322/caac.21834. 38572751 10.3322/caac.21834

[2] Yadav G, Srinivasan G, Jain A. Cervical cancer: Novel treatment strategies offer renewed optimism. Pathol Res Pract. 2024;254:155136. 10.1016/j.prp.2024.155136. 38271784 10.1016/j.prp.2024.155136

[3] Han B, Zheng R, Zeng H, et al. Cancer incidence and mortality in China, 2022. J Natl Cancer Cent. 2024;4:47–53. 10.1016/j.jncc.2024.01.006. 39036382 10.1016/j.jncc.2024.01.006 PMC11256708

[4] Abu-Rustum NR, Campos SM, Amarnath S, et al. Cervical Cancer, Version 2.2026, NCCN Clinical Practice Guidelines In Oncology. J Natl Compr Canc Netw. 2025;23:549–73. 10.6004/jnccn.2025.0057. 41671440 10.6004/jnccn.2025.0057

[5] Francoeur AA, Monk BJ, Tewari KS. Treatment advances across the cervical cancer spectrum. Nat Rev Clin Oncol. 2025;22:182–99. 10.1038/s41571-024-00977-w. 39753753 10.1038/s41571-024-00977-w

[6] Vergote I, González-Martín A, Fujiwara K, et al. Tisotumab Vedotin as Second- or Third-Line Therapy for Recurrent Cervical Cancer. N Engl J Med. 2024;391:44–55. 10.1056/NEJMoa2313811. 38959480 10.1056/NEJMoa2313811

[7] Tewari KS, Monk BJ, Vergote I, et al. Survival with Cemiplimab in Recurrent Cervical Cancer. N Engl J Med. 2022;386:544–55. 10.1056/NEJMoa2112187. 35139273 10.1056/NEJMoa2112187

[8] Anastasio MK, Shuey S, Davidson BA. Antibody-Drug Conjugates in Gynecologic Cancers. Curr Treat Options Oncol. 2024;25:1–19. 10.1007/s11864-023-01166-0. 38172449 10.1007/s11864-023-01166-0

[9] Liu T, Liu Y, Bao X, et al. Overexpression of TROP2 predicts poor prognosis of patients with cervical cancer and promotes the proliferation and invasion of cervical cancer cells by regulating ERK signaling pathway. PLoS ONE. 2013;8:e75864. 10.1371/journal.pone.0075864. 24086649 10.1371/journal.pone.0075864 PMC3785439

[10] Bardia A, Messersmith WA, Kio EA, et al. Sacituzumab govitecan, a Trop-2-directed antibody-drug conjugate, for patients with epithelial cancer: final safety and efficacy results from the phase I/II IMMU-132-01 basket trial. Ann Oncol. 2021;32:746–56. 10.1016/j.annonc.2021.03.005. 33741442 10.1016/j.annonc.2021.03.005

[11] Bardia A, Hurvitz SA, Tolaney SM, et al. Sacituzumab Govitecan in Metastatic Triple-Negative Breast Cancer. N Engl J Med. 2021;384:1529–41. 10.1056/NEJMoa2028485. 33882206 10.1056/NEJMoa2028485

[12] Syed YY. Sacituzumab Govitecan: First Approval. Drugs. 2020;80:1019–25. 10.1007/s40265-020-01337-5. 32529410 10.1007/s40265-020-01337-5 PMC7288263

[13] Zeybek B, Manzano A, Bianchi A, et al. Cervical carcinomas that overexpress human trophoblast cell-surface marker (Trop-2) are highly sensitive to the antibody-drug conjugate sacituzumab govitecan. Sci Rep. 2020;10:973. 10.1038/s41598-020-58009-3. 31969666 10.1038/s41598-020-58009-3 PMC6976591

[14] An J, Li G, Zhang Y, et al. Sacituzumab govitecan for Chinese patients with recurrent/metastatic cervical cancer: Interim analysis of the phase II basket study EVER-132-003. Gynecol Oncol. 2024;190:S22. 10.1016/j.ygyno.2024.07.038. 10.1016/j.ygyno.2025.09.001 40972504

[15] Nie C, Xu W, Guo Y, et al. Immune checkpoint inhibitors enhanced the antitumor efficacy of disitamab vedotin for patients with HER2-positive or HER2-low advanced or metastatic gastric cancer: a multicenter real-world study. BMC Cancer. 2023;23:1239. 10.1186/s12885-023-11735-z. 38102538 10.1186/s12885-023-11735-z PMC10724908

[16] Yin L, Zhou S, Zhang H, et al. Reprogramming the tumor microenvironment: synergistic mechanisms of antibody-drug conjugates and immune checkpoint inhibitors. Antib Ther. 2025;8:262–74. 10.1093/abt/tbaf017. 40989107 10.1093/abt/tbaf017 PMC12451267

[17] Marabelle A, Le DT, Ascierto PA, et al. Efficacy of Pembrolizumab in Patients With Noncolorectal High Microsatellite Instability/Mismatch Repair-Deficient Cancer: Results From the Phase II KEYNOTE-158 Study. J Clin Oncol. 2020;38:1–10. 10.1200/JCO.19.02105. 31682550 10.1200/JCO.19.02105 PMC8184060

[18] Tolaney SM, Cardillo TM, Chou C-C, et al. The Mode of Action and Clinical Outcomes of Sacituzumab Govitecan in Solid Tumors. Clin Cancer Res. 2025;31:1390–9. 10.1158/1078-0432.CCR-24-1525. 39903492 10.1158/1078-0432.CCR-24-1525 PMC11995006

[19] Cardillo TM, Govindan SV, Sharkey RM, et al. Humanized anti-Trop-2 IgG-SN-38 conjugate for effective treatment of diverse epithelial cancers: preclinical studies in human cancer xenograft models and monkeys. Clin Cancer Res. 2011;17:3157–69. 10.1158/1078-0432.CCR-10-2939. 21372224 10.1158/1078-0432.CCR-10-2939 PMC10766325

[20] Wang J, An R, Huang Y, et al. 716MO Efficacy and safety of sacituzumab tirumotecan (sac-TMT) plus pembrolizumab in patients with recurrent or metastatic cervical cancer. Ann Oncol. 2024;35:S548–9. 10.1016/j.annonc.2024.08.778.

[21] MK-2870-020. http://www.chinadrugtrials.org.cn/index.html. Accessed 11 Aug 2025.

[22] BAT-8008-001-CR. http://www.chinadrugtrials.org.cn/index.html. Accessed 11 Aug 2025.

[23] Lopez S, Perrone E, Bellone S, et al. Preclinical activity of sacituzumab govitecan (IMMU-132) in uterine and ovarian carcinosarcomas. Oncotarget. 2020;11:560–70. 10.18632/oncotarget.27342. 32082489 10.18632/oncotarget.27342 PMC7007291

[24] Chung HC, Ros W, Delord J-P, et al. Efficacy and Safety of Pembrolizumab in Previously Treated Advanced Cervical Cancer: Results From the Phase II KEYNOTE-158 Study. J Clin Oncol. 2019;37:1470–8. 10.1200/JCO.18.01265. 30943124 10.1200/JCO.18.01265

[25] Naumann RW, Hollebecque A, Meyer T, et al. Safety and Efficacy of Nivolumab Monotherapy in Recurrent or Metastatic Cervical, Vaginal, or Vulvar Carcinoma: Results From the Phase I/II CheckMate 358 Trial. J Clin Oncol. 2019;37:2825–34. 10.1200/JCO.19.00739. 31487218 10.1200/JCO.19.00739 PMC6823884

[26] Zheng X, Gu H, Cao X, et al. Tislelizumab for cervical cancer: A retrospective study and analysis of correlative blood biomarkers. Front Immunol. 2023;14:1113369. 10.3389/fimmu.2023.1113369. 36875089 10.3389/fimmu.2023.1113369 PMC9975598

[27] Grivas P, Pouessel D, Park CH, et al. Sacituzumab Govitecan in Combination With Pembrolizumab for Patients With Metastatic Urothelial Cancer That Progressed After Platinum-Based Chemotherapy: TROPHY-U-01 Cohort 3. J Clin Oncol. 2024;42:1415–25. 10.1200/JCO.22.02835. 38261969 10.1200/JCO.22.02835 PMC11095901

[28] Reck M, Patel JD, Gray JE, et al. First-Line Sacituzumab Govitecan Plus Pembrolizumab in Metastatic NSCLC: PD-L1 TPS < 50% and ≥ 50% Cohorts of the EVOKE-02 Study. J Thorac Oncol. 2025;S1556–0864(25):02890–4. 10.1016/j.jtho.2025.10.016. 10.1016/j.jtho.2025.10.016 41173143

[29] Fang W, Wang Q, Cheng Y, et al. Sacituzumab tirumotecan (sac-TMT) in combination with tagitanlimab (anti-PD-L1) in first-line (1L) advanced non-small-cell lung cancer (NSCLC): Non-squamous cohort from the phase II OptiTROP-Lung01 study. JCO. 2025;43:8529–8529. 10.1200/JCO.2025.43.16_suppl.8529.

[30] Wei Y, Zhang R, Yu C, et al. Disitamab vedotin in combination with immune checkpoint inhibitors for locally and locally advanced bladder urothelial carcinoma: a two-center’s real-world study. Front Pharmacol. 2023;14:1230395. 10.3389/fphar.2023.1230395. 37645442 10.3389/fphar.2023.1230395 PMC10461006

[31] Gu H, Zhou J, Wang J, et al. PT002/#568 Cadonilimab in combination with disitamab vedotin or nab-paclitaxel in the treatment of recurrent or metastatic cervical cancer: a prospective, double-cohort, multi-center, open-label, phase II clinical study (AK001). Int J Gynecol Cancer. 2024;34:A23–4. 10.1136/ijgc-2024-IGCS.25.

[32] O’Donnell PH, Milowsky MI, Petrylak DP, et al. Enfortumab Vedotin With or Without Pembrolizumab in Cisplatin-Ineligible Patients With Previously Untreated Locally Advanced or Metastatic Urothelial Cancer. J Clin Oncol. 2023;41:4107–17. 10.1200/JCO.22.02887. 37369081 10.1200/JCO.22.02887 PMC10852367

