# Supplementary material for: Sacituzumab govitecan combined with tislelizumab as second-line therapy for recurrent or metastatic cervical cancer: a case report and literature review
Source: BMC Womens Health. 2026 Apr 22;26:284. doi: 10.1186/s12905-026-04481-4 (PMC13235119; doi:10.1186/s12905-026-04481-4)
Supplement: Supplementary file 2 — Supplementary Material 2. [file 12905_2026_4481_MOESM2_ESM.docx]

PubMed advanced search query:

(("Immune Checkpoint Inhibitors"[MeSH] OR "Immune Checkpoint Blockade"[tiab] OR "Immune Checkpoint Inhibitor*"[tiab] OR "Immune Checkpoint Blocker*"[tiab] OR "Immune Checkpoint Inhibition"[tiab] OR "PD-L1 Inhibitor*"[tiab] OR "PD-1 Inhibitor*"[tiab] OR "CTLA-4 Inhibitor*"[tiab] OR "PD-1-PD-L1 Blockade"[tiab] OR "pembrolizumab"[tiab] OR "nivolumab"[tiab] OR "cemiplimab"[tiab] OR "dostarlimab"[tiab] OR "tislelizumab"[tiab] OR "sintilimab"[tiab] OR "camrelizumab"[tiab] OR "toripalimab"[tiab] OR "atezolizumab"[tiab] OR "avelumab"[tiab] OR "durvalumab"[tiab] OR "sugemalimab"[tiab] OR "envafolimab"[tiab] OR "ipilimumab"[tiab] OR "tremelimumab"[tiab] OR "relatlimab"[tiab])) AND ((("TROP2"[tiab] OR "Trop-2"[tiab] OR "TACSTD2"[tiab] OR "EGP-1"[tiab]) AND ("Immunoconjugates"[MeSH] OR "Antibody-Drug Conjugate*"[tiab] OR "ADC"[tiab] OR "ADCs"[tiab])) OR ("sacituzumab govitecan"[tiab] OR "Trodelvy"[tiab] OR "IMMU-132"[tiab] OR "datopotamab deruxtecan"[tiab] OR "Dato-DXd"[tiab] OR "DS-1062"[tiab] OR "SKB264"[tiab] OR "MK-2870"[tiab]))
